# Dialogues in Immunity: The Interplay Between Neutrophils and Macrophages

**DOI:** 10.3390/biomedicines14030520

**Published:** 2026-02-26

**Authors:** Eduardo Anitua, María Troya, Mohammad H. Alkhraisat

**Affiliations:** 1 BTI-Biotechnology Institute, 01007 Vitoria, Spain; maria.troya@bti-implant.es (M.T.); mohammad.hamdan@bti-implant.es (M.H.A.); 2 University Institute for Regenerative Medicine & Oral Implantology, UIRMI (UPV/EHU-Fundación Eduardo Anitua), 01007 Vitoria, Spain; 3 Oral and Maxillofacial Surgery, Oral Medicine and Periodontics Department, Faculty of Dentistry, University of Jordan, Amman 11942, Jordan

**Keywords:** neutrophils, macrophages, immunity, regenerative medicine

## Abstract

Cooperation between neutrophils and macrophages is essential to innate immunity. Though they share origins, their distinct roles make them complementary in fighting pathogens and regulating inflammation. However, dysregulation can drive chronic inflammation and autoimmune disease, making therapeutic targeting highly challenging. Broad suppression of these cells is risky; instead, precision strategies are needed to modulate their dual roles in promoting and resolving inflammation. Aging further complicates this balance, as impaired neutrophil and macrophage functions—alongside microbiota alterations—contribute to inflammaging and immune dysfunction. Recent advances in technology offer opportunities to explore these interactions in physiologically relevant contexts, paving the way for targeted interventions that restore immune homeostasis without compromising defense mechanisms. This article highlights the need for nuanced approaches to harness neutrophil–macrophage cooperation for therapeutic benefit.

## 1. Introduction

**Figure 1:**
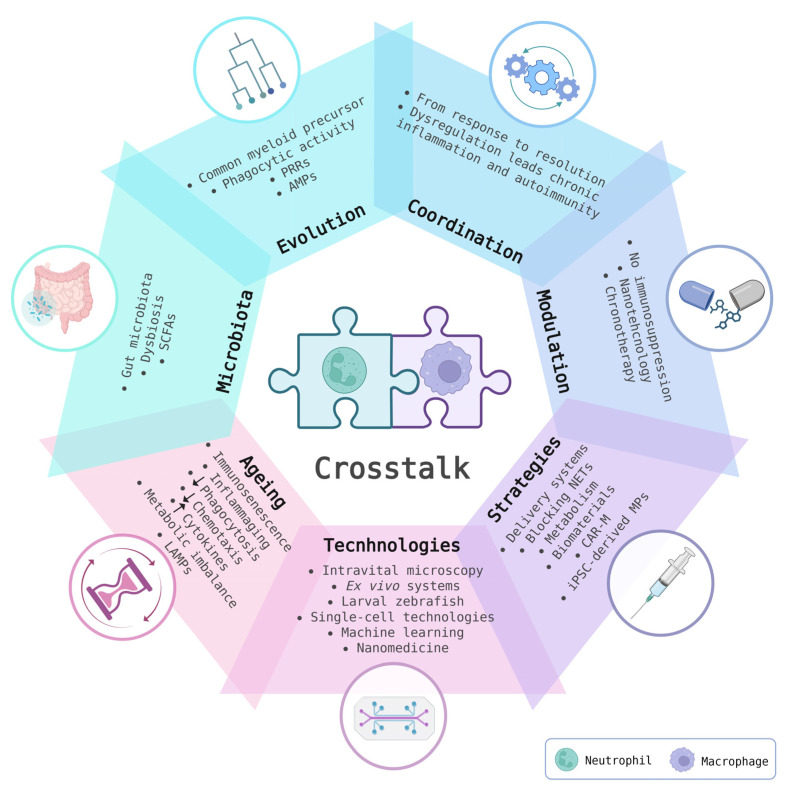
Neutrophil–Macrophage crosstalk as a central axis in innate immunity. The figure presents a conceptual synthesis of seven key dimensions through which neutrophil–macrophage interactions shape innate immune responses and therapeutic strategies: (1) their evolutionary conservation and specialization; (2) coordinated innate immune functions; (3) potential for targeted therapeutic modulation; (4) expanding the focus beyond lymphocytes; (5) insights from advanced experimental models; (6) the influence of ageing and immunosenescence on their behaviour; and (7) the influence of microbiota-immune system crosstalk. These interconnected aspects highlight the central role of neutrophil–macrophage crosstalk in both physiological and pathological contexts. Created in BioRender. Anitua, E. (2026) https://BioRender.com/01cvyis (accessed on 18 February 2026). AMPs: antimicrobial peptides; CAR-M: chimeric antigen receptor macrophages; iPSC: induced pluripotent stem cell; LAMPs: lifestyle-associated molecular patterns; MPs: macrophages; PRR: pattern recognition receptors; SCFAs: short-chain fatty acids.

The cooperation between neutrophils and macrophages represents a cornerstone of innate immunity, shaped by millions of years of evolution to ensure effective pathogen elimination, tissue repair, and resolution of inflammation. From the perspective of regenerative medicine, this interaction can be explored through seven major domains (Figure 1): evolutionary development, functional coordination, therapeutic potential modulation, therapeutic strategies, experimental models, immunosenescence, and microbiota. These two phagocytic cells originate from common myeloid progenitors and share essential features such as potent phagocytic activity, expression of pattern recognition receptors (PRRs), and production of antimicrobial peptides (AMPs)—all of which are highly conserved across phylogeny [1,2]. The origins of innate immunity trace back to 1882 when Elie Metchnikoff identified myeloid-like cells in starfish larvae responding to a foreign object, demonstrating that cellular immune responses are deeply rooted even in primitive organisms [3]. Despite their shared origin and overlapping functions, neutrophils and macrophages exhibit distinct morphological and functional specializations that make them complementary and irreplaceable in host defence. Evolutionary conservation is interlaced with species-specific adaptations tailored to diverse ecological niches, reflecting the dynamic specialization of immune systems. For example, single-cell RNA sequencing and transcriptomic analyses have shown that macrophage genes involved in phagocytosis and degradation of foreign material, such as cathepsins, are evolutionarily conserved, whereas chemokine families like the C–C motif chemokine ligands (CCLs), which mediate cell migration, display notable evolutionary divergence [4]. Furthermore, in silico analyses have linked neutrophil development and functional dominance to the evolution of CSF3R/CSF3 signalling, with significant roles emerging in tetrapods. The advent of endothermy further enhanced the prevalence and specialization of neutrophils/heterophils in mammals and birds [5]. The interplay between neutrophils and macrophages represents a multifaceted relationship deeply involved in tissue repair processes. The clinical relevance of the neutrophil–macrophage axis is best illustrated by diseases where their interaction is fundamentally disrupted. For example, in chronic granulomatous disease (CGD), impaired NADPH oxidase function disrupts both neutrophil apoptosis clearance and macrophage efferocytosis, leading to persistent neutrophil accumulation, unresolved inflammation, and autoimmunity [6]. Similarly, in cystic fibrosis (CF), excessive neutrophil elastase released into the airway profoundly impairs macrophage phagocytosis and phagolysosome formation, globally altering macrophage function [7]. In both conditions, dysfunctional neutrophil–macrophage interactions drive defective clearance mechanisms and chronic inflammation.

This article seeks to highlight the reparative and regulatory roles of neutrophils and macrophages and the cooperative interactions that emerge between them, moving beyond the traditional view that confines these cells to pathogenic mechanisms and cytotoxic functions. While these detrimental aspects remain relevant for therapeutic regulation and strategy design, the complexity of these processes requires precise control to achieve beneficial outcomes while avoiding harmful side effects. The convergence of these dual mechanisms creates an intricate biological landscape. A deeper understanding of their dynamic roles could unlock new therapeutic avenues.

## 2. Functional Coordination

**Figure 2:**
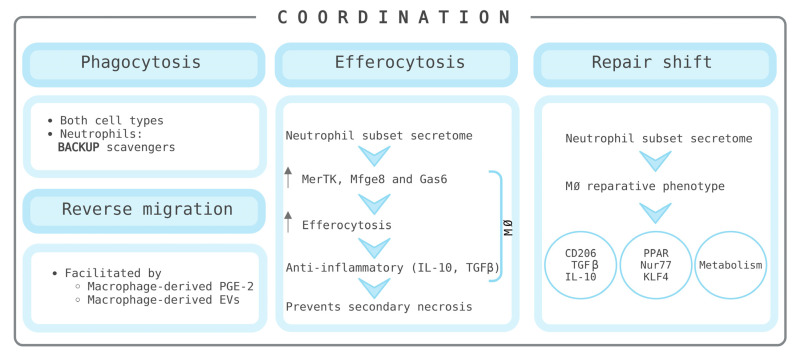
Main processes through which neutrophils cooperate with macrophages to promote tissue repair, including phagocytosis, efferocytosis, macrophage reprogramming toward a reparative state, and neutrophil reverse migration. Created in BioRender. Anitua, E. (2026) https://BioRender.com/hve35uv (accessed on 18 February 2026) EVs: extracellular vesicles; MØ: macrophages; PGE-2: prostaglandin E2.

Coordination between neutrophils and macrophages ensures efficient pathogen elimination and inflammation control, with neutrophils acting as a backup when macrophage scavenging is overwhelmed (Figure 2). Their cooperation enhances innate immune responses through shared receptor expression and cytokine signalling and is crucial for orchestrating adaptive immunity and maintaining homeostasis. Moreover, neutrophils and macrophages orchestrate the transition from the acute response to resolution. However, dysregulation in their functions can lead to chronic inflammation and contribute to the pathogenesis of autoimmune diseases. Neutrophils, traditionally viewed as short-lived pro-inflammatory cells, are now recognized as a heterogeneous population with distinct functional subsets. Among these, anti-inflammatory N2 neutrophils play a pivotal role in promoting resolution. They secrete factors that induce a reparative macrophage phenotype, characterized by the upregulation of anti-inflammatory markers such as CD206, TGF-β, and IL-10, as well as nuclear regulators like PPAR, Nur77, and KLF4. These neutrophils also enhance efferocytosis—the process by which macrophages engulf apoptotic cells—by increasing the expression of receptors such as MerTK, Mfge8, and Gas6 [8]. Efferocytosis is a key mechanism in the transition from inflammation to resolution. When macrophages engulf apoptotic neutrophils, they shift toward an anti-inflammatory phenotype and secrete cytokines like IL-10 and TGF-β, which suppress further immune activation and promote tissue healing. This process also prevents secondary necrosis, which would otherwise release pro-inflammatory cytokines and reactive oxygen species (ROS), exacerbating tissue damage. At the same time, emerging evidence shows that efferocytosis is not uniformly efficient across all physiological contexts. Dysregulated efferocytosis in metabolic diseases such as obesity and diabetes can perpetuate chronic inflammation by altering macrophage polarization, reducing phagocytic efficiency, and impairing tissue-repair programs. Impaired TAM-receptor signaling further disrupts the metabolic reprogramming that normally follows apoptotic-cell uptake, thereby prolonging inflammation and delaying regeneration. When TAM receptors are dysfunctional or absent, apoptotic cells accumulate and become immunogenic, triggering aberrant immune activation and self-directed inflammatory responses [9]. Another resolution mechanism is reverse migration, where neutrophils exit inflamed tissues and re-enter circulation, potentially undergoing clearance in the lungs or bone marrow. This process is facilitated by macrophage-derived prostaglandin E2. In chronic wounds, impaired resolution is associated with persistent neutrophil infiltration, excessive ROS and protease activity, and reduced levels of tissue inhibitors of metalloproteinases (TIMPs), all of which hinder healing [10,11]. The liver provides a compelling example of this coordinated immune response. During liver regeneration, neutrophils and Kupffer cells (liver-resident macrophages) engage in mutual recruitment via chemokines such as Cxcl2, highlighting the importance of their interaction in tissue repair [12]. Neutrophils also promote a phenotypic shift in macrophages from a pro-inflammatory (Ly6C^hi^CX3CR1^lo^) to a pro-resolving (Ly6C^lo^CX3CR1^hi^) state, a transition driven, among others, by neutrophil-derived ROS and potentially mediated by AMPK activation, which reprograms macrophage metabolism toward a reparative profile [13]. This transition represents a tightly regulated reprogramming of redox-sensitive pathways rather than a simple phenotypic shift. Mechanistically, ROS disrupt the Nrf2-Keap1 complex by oxidizing critical cysteine residues on Keap1, thereby preventing Nrf2 degradation and facilitating its nuclear translocation. Once active, Nrf2 orchestrates the expression of antioxidant and anti-inflammatory genes while concurrently inhibiting NF-κB signaling [14,15]. This integrated modulation—encompassing the Nrf2, AMPK, and NF-κB axes—ensures the silencing of pro-inflammatory loci and executes a metabolic shift that primes the macrophage for tissue repair [16].

Given their dual roles in both promoting and resolving inflammation, neutrophils and macrophages are attractive targets for therapeutic intervention. However, broad suppression of their activity poses risks, as these cells are essential for host defence and tissue repair.

## 3. Therapeutic Modulation

Therapeutic strategies are increasingly focused on modulating specific functions rather than inducing global immunosuppression (Table 1). Nanotechnology offers a powerful platform for targeted drug delivery. Nanoparticles can be engineered to bind selectively to neutrophils or macrophages, modulating their activation, migration, and phenotype. This precision aims to reduce systemic side effects and enhance therapeutic efficacy. For example, nanoparticles can deliver anti-inflammatory agents directly to inflamed tissues or promote the polarization of macrophages toward a reparative M2 phenotype [17,18]. Nevertheless, translating this potential requires overcoming significant physiological barriers, such as nonspecific uptake by the liver and spleen, reticuloendothelial system (RES) sequestration, and unpredictable biodistribution–factors that continue to limit therapeutic precision [19]. Acknowledging these hurdles is essential for putting current enthusiasm into proper context. Chronopharmacology, which aligns drug administration with circadian rhythms, is another emerging strategy. Neutrophils exhibit circadian regulation of their granule content and NET-forming capacity, governed by CXCR2 signaling and clock genes like Bmal1. This intrinsic “disarming” program reduces neutrophil-mediated tissue damage in highly vascularized organs such as the lungs. Therapeutic interventions that leverage these temporal dynamics may enhance resolution while minimizing inflammation-related injury [20].

## 4. Beyond Lymphocytes

Current immunotherapy largely targets lymphocytes, such as CAR-T cells, but emerging strategies are also focusing on macrophage reprogramming and neutrophil modulation (Table 1). Therapeutic strategies that promote the shift of macrophages from the inflammatory M1 to the reparative M2 phenotype —through drug delivery systems, nanoparticles, or cell transplantation—can reduce inflammation and promote tissue regeneration. Delivery systems targeting macrophages include ligand-guided drugs, macrophage-based carriers, and extracellular vesicles [21]. In parallel, neutrophil activity is being refined: once broadly suppressed, neutrophils are now precisely regulated to resolve inflammation without compromising immunity. This is achieved through pro-resolving mediators, receptor targeting (e.g., Mac-1, ALX/FPR2) [22], and interventions that inhibit chemotaxis, ROS production, and key signaling pathways via CXCR2 antagonists and JAK inhibitors [17,23,24]. Additionally, blocking neutrophil extracellular trap (NET) formation—particularly via PAD4 inhibition—has shown promise in reducing tissue damage and immune activation [17,23,24]. Still, therapeutic PAD4 inhibition must be approached cautiously, as NETs are fundamental to host defense. Beyond increasing susceptibility to opportunistic infections, PAD4 also shapes immune regulation, affecting antigen presentation and cytokine function. Consequently, its inhibition may simultaneously attenuate tissue-damaging inflammation while potentially compromising protective host responses [25]. These trade-offs require precise dosing or site-specific delivery to preserve overall immune competence. Several FDA-approved drugs have demonstrated the ability to modulate macrophage polarization and neutrophil activity. Small molecules can inhibit pro-inflammatory M1 macrophages, promote M2-like polarization, or induce M1-to-M2 repolarization—strategies used to treat arthritis, colitis, and diabetes-related conditions [21]. Brensocatib, a dipeptidyl peptidase 1 inhibitor—an enzyme responsible for activating neutrophil serine proteases—was recently approved for non-cystic fibrosis bronchiectasis and offers a novel anti-inflammatory approach targeting neutrophil-driven disease [26]. Macrophage and neutrophil plasticity are closely linked to their metabolic states, with the CSF1/CSF1R axis playing a key role in macrophage regeneration [27,30]. Biomaterials like hydrogels are being designed to guide macrophage polarization, while advanced therapies such as CAR-M (chimeric antigen receptor macrophages) and iPSC-derived macrophages integrate immune engineering with precision delivery [27]. However, the clinical translation of CAR-M remains in its infancy, with limited human data and significant hurdles posed by the immunosuppressive pathological microenvironment, where factors such as hypoxia, inhibitory cytokines, and metabolic constraints can impair their persistence and activity [28]. Overcoming these barriers is essential for further optimization and for preventing CAR-M exhaustion. In acute organ injuries (AOIs), where inflammation and mitochondrial dysfunction are prevalent, novel therapies like nMITO—which combines neutrophil membranes with mitochondria—offer dual-action treatment by targeting inflammation and promoting cellular repair [29].

## 5. Advanced Experimental Models

In recent years, the study of immune cell interactions—particularly between macrophages and neutrophils—has entered a new era, driven by a wave of technological innovation. Traditional models often fell short in replicating the complexity of human biology, but now, researchers are equipped with tools that allow them to explore these interactions in environments that closely mimic real tissues and disease conditions. Tools like intravital microscopy (IVM)—especially confocal and two-photon imaging— enables real-time, high-resolution visualization of immune cell behaviour in living organisms, offering insights into their migration, interaction, and function across various organs [31]. While powerful in animal models, human IVM is limited to accessible vasculature, therefore, researchers have turned to ex vivo systems—such as microfluidic devices, organoids, and organ-on-chip platforms—to simulate the human immune environment more precisely. Organoids, especially when co-cultured with macrophages and integrated with microfluidics, offer realistic simulations of human biology for drug testing and disease modelling [32,33]. Larval zebrafish represent a powerful model for studying innate immunity due to their transparent bodies, conserved immune system (similar to humans), and undeveloped adaptive immunity during early stages. This and their genetic tractability make them ideal for immunological research [34]. Transgenic mouse models also provide valuable in vivo platforms due to their genetic accessibility. However, findings should be cautiously extrapolated to other species due to interspecies immune differences. As neutrophil depletion is not feasible in most human conditions, the focus has shifted to identifying targetable pathogenic subsets. This effort is supported by single-cell technologies, including transcriptomics, proteomics, and epigenomics, which reveal the remarkable heterogeneity and plasticity of these immune cells [35]. Importantly, these high-resolution human datasets are now being integrated with clinical trial data to validate whether the expansion of specific subsets—such as those identified in inflammatory bowel disease—can serve as biomarkers for disease severity or therapeutic response [36]. By integrating machine learning and nanomedicine, macrophage polarization and behaviour can also be tracked using nanoscale probes that enable non-invasive, real-time imaging—offering powerful tools for optimizing therapeutic strategies [37].

## 6. Ageing and Immunesenescence

Aging profoundly alters the immune system, particularly the coordination between neutrophils and macrophages, leading to weakened immune responses and persistent low-grade inflammation, known as inflammaging. In older adults, neutrophils display reduced efficiency in chemotaxis, phagocytosis, and microbial killing [38,39]. Macrophages similarly exhibit age-associated metabolic dysfunctions. These impairments are also evident in monocyte-derived macrophages, which show diminished phagocytic capacity, migratory ability, and chemotactic response. Such functional declines are linked to the downregulation of transcription factors MYC and USF1 [40]. This disruption in cellular communication and function contributes to prolonged inflammation, impaired tissue repair, and increased vulnerability to infections and age-related chronic diseases. The broader decline in immune function, termed immunosenescence, affects both innate and adaptive immunity. It weakens immune memory and vaccine responses, increasing the risk of chronic diseases, including cardiovascular diseases, metabolic disorders, autoimmune diseases and neurodegenerative diseases [41]. Key contributors include the accumulation of senescent cells, imbalance in immune cell populations, and gut microbiome dysbiosis, all of which promote systemic inflammation and metabolic decline. Senescent cells, which permanently exit the cell cycle due to stress, release inflammatory molecules known as the senescence-associated secretory phenotype (SASP). SASP perpetuates inflammation and disrupts immune regulation [38]. Aging disrupts key immune functions, beginning with impaired macrophage metabolism and a reduced ability to clear apoptotic cells, which prolongs inflammation. Neutrophils also become less effective in eliminating pathogens and supporting wound healing, as changes in reactive oxygen species (ROS) production and neutrophil extracellular trap (NET) formation contribute to tissue damage. This immune dysregulation is accompanied by systemic inflammation, reflected in elevated levels of cytokines such as IL-6 and TNF-α. Additionally, neutrophil migration slows and loses directionality, further delaying wound repair and leading to excessive accumulation in aged tissues [38,42]. In skeletal muscle, these inflammatory and oxidative changes accelerate the loss of muscle mass and function, highlighting the broader impact of aging on tissue integrity and immune response [43]. Environmental exposures—collectively known as the exposome—including diet, pollution, and stress, influence immune aging through lifestyle-associated molecular patterns (LAMPs). These factors can accelerate or mitigate immune decline [44]. Promising therapeutic strategies include senolytics, SASP inhibitors, immune modulators, and microbiome-targeted therapies, which aim to restore immune balance and promote healthy aging [41].

## 7. Microbiota-Immune Crosstalk

As humans age, the gut microbiota—an essential regulator of immune responses—also experiences significant changes, contributing to this age-related immune imbalance. The gut microbiota, often described as a “superorganism,” plays a central role in shaping both innate and adaptive immunity. In a state of eubiosis, it supports immune tolerance, epithelial integrity, and pathogen defence. However, aging, poor diet, antibiotic use, and disease can lead to dysbiosis—an imbalance in microbial populations. This condition increases intestinal permeability, allowing microbial antigens to enter the bloodstream and activate immune receptors such as toll-like receptors (TLRs), thereby promoting chronic inflammation [45]. Neutrophils and macrophages are influenced by microbiota-derived metabolites, particularly short-chain fatty acids (SCFAs) like butyrate, acetate, and propionate. These SCFAs regulate immune cell activity through G-protein–coupled receptors and histone deacetylase inhibition, modulating reactive oxygen species (ROS) production and inflammatory resolution [46,47]. In inflammatory conditions such as ulcerative colitis (UC), dysbiosis reduces SCFA levels, impairing neutrophil regulation and perpetuating inflammation [47]. Neutrophils also shape the microbiota by releasing antimicrobial peptides (AMPs), including defensins and cathelicidins. These peptides help maintain microbial balance and mucosal protection. For instance, mice lacking CRAMP—a cathelicidin—exhibit gut dysbiosis and increased susceptibility to colitis, highlighting the reciprocal influence between immune cells and microbiota [46]. Emerging therapies aim to restore microbial balance and immune regulation through probiotics, prebiotics, fecal microbiota transplantation (FMT), and dietary phenolic acids. The latter compounds, like chlorogenic and ferulic acid, modulate immune responses and reduce inflammation by targeting macrophage polarization and NET formation [48]. Ultimately, the gut microbiota’s influence extends beyond local immunity, affecting systemic functions such as metabolism, circadian rhythms, and neurological health. Its bidirectional interaction with the immune system is crucial for maintaining health, especially in the context of aging and immunosenescence.

## 8. Concluding Remarks

The interplay between neutrophils and macrophages exemplifies a multifaceted relationship central to tissue repair. A deeper understanding of their dynamic roles could unlock novel therapeutic opportunities. However, this approach faces significant challenges due to the inherent therapeutic paradox: immunosuppression is not viable, as it increases infection risk and compromises patient safety, while complete suppression of inflammation is equally problematic because early inflammatory responses are essential for wound healing. Therefore, rather than broadly suppressing immune activity, therapeutic strategies should aim to modulate specific functions that promote resolution pathways. Coordinated activity between neutrophils and macrophages ensures effective pathogen clearance and inflammation control, making this interaction an attractive target for intervention—despite its underlying heterogeneity. In this sense, neutrophil and macrophage behavior exhibits remarkable tissue-specific variability, complicating the development of generalized therapeutic strategies. Recruitment mechanisms differ not only across organs but also within the same tissue under distinct inflammatory stimuli. Innate immune cells rapidly adapt to local microenvironments and acquire distinct phenotypic and functional profiles [49,50]. This heterogeneity underscores the need for tailored, tissue-specific interventions rather than universal approaches, as precise modulation of these interactions is essential to balance repair, defense, and inflammation without compromising host immunity. On the other hand, the M1/M2 macrophage paradigm—and its N1/N2 analogue in neutrophils—represents an oversimplification of their true complexity. These highly plastic cells exist along a continuum of activation states, exhibiting overlapping phenotypes shaped by tissue context and the specific signals they encounter [51]. Recognizing these limitations, a key objective is to elucidate the signaling pathways and transcriptional networks that govern their dynamic reprogramming.

Future therapeutic efforts should embrace integrative approaches, including nanotechnology, chronotherapy, advanced drug delivery systems, cell transplantation, biomaterials, and biologic strategies capable of reprogramming and fine-tuning neutrophil–macrophage interactions. Within this spectrum of biologic interventions, platelet-derived products offer a particularly illustrative example of how additional innate immune partners can modulate this axis. Platelets are now recognized as key regulators of innate immunity, interacting closely with neutrophils and macrophages through a broad repertoire of immune receptors. Their dynamic crosstalk not only drives thrombosis and inflammation but also contributes to inflammation resolution, tissue repair, and wound healing [52,53,54]. Platelet-rich plasma (PRP), known for its regenerative potential, leverages these immunomodulatory properties by orchestrating a timely shift in macrophage polarization (M1–M2) and modulating native neutrophil activity during tissue repair [55,56,57,58,59]. However, the lack of standardized preparation protocols still results in heterogeneous formulations and variable clinical outcomes. Specifically, the frequent omission of product composition, activation methods, and precise dosage in published studies hinders cross-study comparisons and may lead to contradictory findings. Despite ongoing efforts to enforce standardization [60], this lack of detailed reporting persists, underscoring the need for deeper insight into platelet–immune cell interactions within platelet-enriched products. As these examples highlight, the networks governing neutrophil–macrophage communication are embedded within broader layers of regulation. The interplay among aging, microbiota composition, and immune function further contributes to inflammaging and immune dysfunction, adding complexity to these cellular interactions. Advancing our ability to study such interconnected immune processes through intravital microscopy, ex vivo systems, single cell analysis, and nanomedicine will be essential to capture the remarkable heterogeneity and metabolic plasticity that define these immune cells and their microenvironment, while also acknowledging that species-specific differences in neutrophil–macrophage crosstalk make translation to humans challenging [61]. Incorporating this knowledge into the design of clinical trials is equally important, as their complexity cannot be reproduced in simplified in vitro models, nor adequately account for patient heterogeneity. In this regard, advances in microfluidics will play a key role.

The therapeutic manipulation of the neutrophil–macrophage axis presents a strategic dilemma: which cell type offers the most effective entry point for regenerative medicine? Targeting neutrophils provides a critical ‘upstream’ advantage. Since they are the first responders to injury, modulating their activity can prevent initial collateral damage. Intervening at this stage is essentially a ‘damage control’ strategy that preserves the tissue microenvironment, making it more receptive to subsequent healing signals. Furthermore, as neutrophils provide the instructive signals for macrophage polarization, reprogramming the neutrophil output can indirectly facilitate a more efficient transition toward a pro-resolving state. On the other hand, macrophages offer a broader ‘therapeutic window’ due to their longevity and their direct role in orchestrating the final stages of regeneration. Therefore, neither cell type represents a universally superior therapeutic target; rather, the choice depends on the temporal dynamics and pathological context of inflammation, reinforcing the relevance of understanding their coordinated actions. Ultimately, this integrative framework for dissecting neutrophil–macrophage crosstalk enables researchers to explore immune cells within a dynamic cellular ecosystem, revealing the nuanced interactions between these populations and their surrounding milieu—and paving the way for innovative therapeutic strategies.

## Figures and Tables

**Table 1:** Emerging therapeutic strategies targeting myeloid cell plasticity and function. The table summarizes current and experimental approaches to modulate macrophage and neutrophil activity. CAR-M: chimeric antigen receptor macrophages; DPP1: dipeptidyl peptidase 1; EVs: extracellular vesicles; NET: neutrophil extracellular traps; RES: reticuloendothelial system; ROS: reactive oxygen species.

Therapeutic Strategy	Mechanisms	Limitations & Considerations	References
Nanoparticle-based immune modulation	Targeted drug delivery to neutrophils/macrophages for modulating their activation and phenotype.	▪ Nonspecific uptake by liver/spleen. ▪ RES sequestration. ▪ Unpredictable biodistribution.	[17,18,19]
Chronopharmacology	Aligns therapy with circadian rhythms (via CXCR2 and Bmal1 signalling) to reduce neutrophil-mediated damage.	▪ Limited clinical validation. ▪ Clinical administration complexity. ▪ Timing/dosing challenges.	[20]
Macrophage reprogramming	Ligand-guided drugs, carriers, EVs, nanoparticles or cell transplantation to shift M1 to M2 phenotype.	▪ Compromising immune response. ▪ Microenvironment dependent.	[21]
Neutrophil modulation	Pro-resolving mediators, receptor targeting, inhibition of chemotaxis and ROS production and signalling pathways via CXCR2 antagonists and JAK inhibitors.	▪ Potential for increased susceptibility to infections. ▪ Narrow therapeutic window.	[17,22,23,24]
NET inhibition	Particularly via PAD4 inhibition, reducing tissue damage and immune activation.	▪ Increasing susceptibility to opportunistic infections. ▪ Effects on antigen presentation and cytokine activity.	[17,23,24,25]
Approved drugs modulating macrophages and neutrophils	Small molecules that suppress M1, promote M2 polarization, or induce M1-to-M2 repolarization; e.g., Brensocatib, a DPP1 inhibitor that reduces neutrophil proteases, providing targeted anti-inflammatory effects in neutrophil-driven disease.	▪ Systemic side effects. ▪ Lack of long-term safety data. ▪ Limited to a few diseases.	[21,26]
CAR-M and iPSC-derived macrophages	Immune engineering with precision delivery.	▪ Clinical translation in infancy. ▪ Limited human data. ▪ Immunosuppressive pathological microenvironment.	[27,28]
nMITO therapy	Neutrophil membranes with mitochondria.	▪ Emergent strategy. ▪ Limited data.	[29]

## Data Availability

No new data were created or analyzed in this study.
